# Supplementary material for: Changes in the transcriptome and synthetic lethal dependencies following KRAS mutant expression reveal profound tissue specificity
Source: Proc Natl Acad Sci U S A. 2026 Sep 2;123(36):e2614664123. doi: 10.1073/pnas.2614664123 (PMC13552922; doi:10.1073/pnas.2614664123)
Supplement: Supplementary file 1 [file pnas.2614664123.sapp.pdf]

PNAS

Supporting Information for

Changes in the Transcriptome and Synthetic Lethal Dependencies Following *KRAS* Mutant Expression Reveal Profound Tissue-Specificity

Timothy D Martin[1,2,#], Mei Yuk Choi[1,2], Julia C McBride[1,2], Stephen J Elledge[1,2,3,*]

1 Howard Hughes Medical Institute, Department of Genetics, Harvard University Medical School, Boston, MA, USA

2 Division of Genetics, Brigham and Women's Hospital, Boston, MA, USA

3 Program in Virology, Harvard University Medical School, Boston, MA, USA

Present Address

Department of Pharmacology, University of Virginia School of Medicine, Charlottesville, VA, USA

*Stephen J Elledge, Corresponding Author

Email: selledge@genetics.med.harvard.edu

This PDF file includes:

Supplemental Figures S1 to S3
Legends for Datasets S1 to S3

Other supporting materials for this manuscript include the following:

Datasets S1 to S3

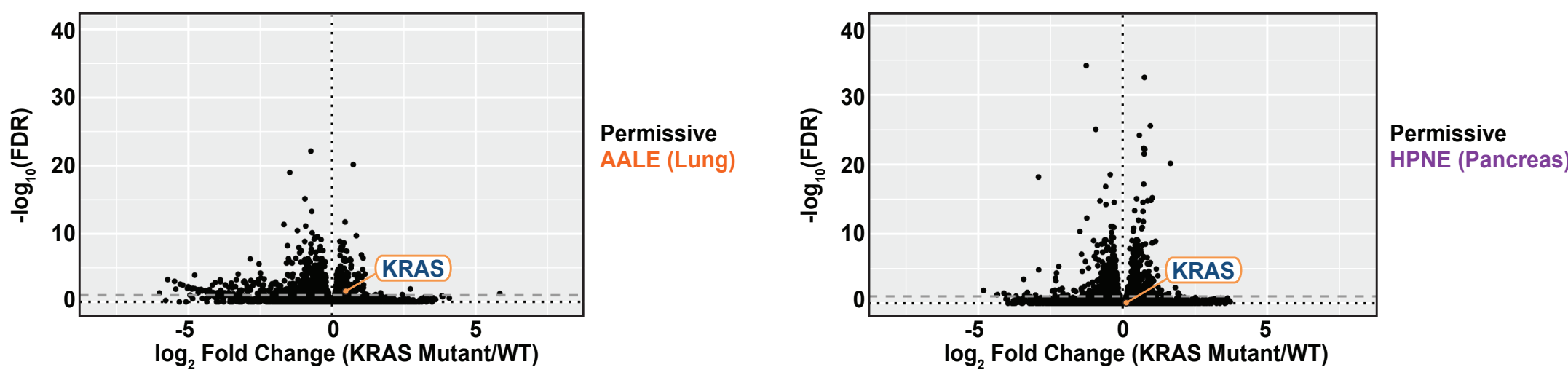

Figure S1. Transcriptome changes in permissive AALE and HPNE cell lines. A. Volcano plots illustrating the magnitude and significance of differentially expressed genes (DEGs) following *KRAS* induction in permissive AALE (left) and HPNE (right) cell lines.

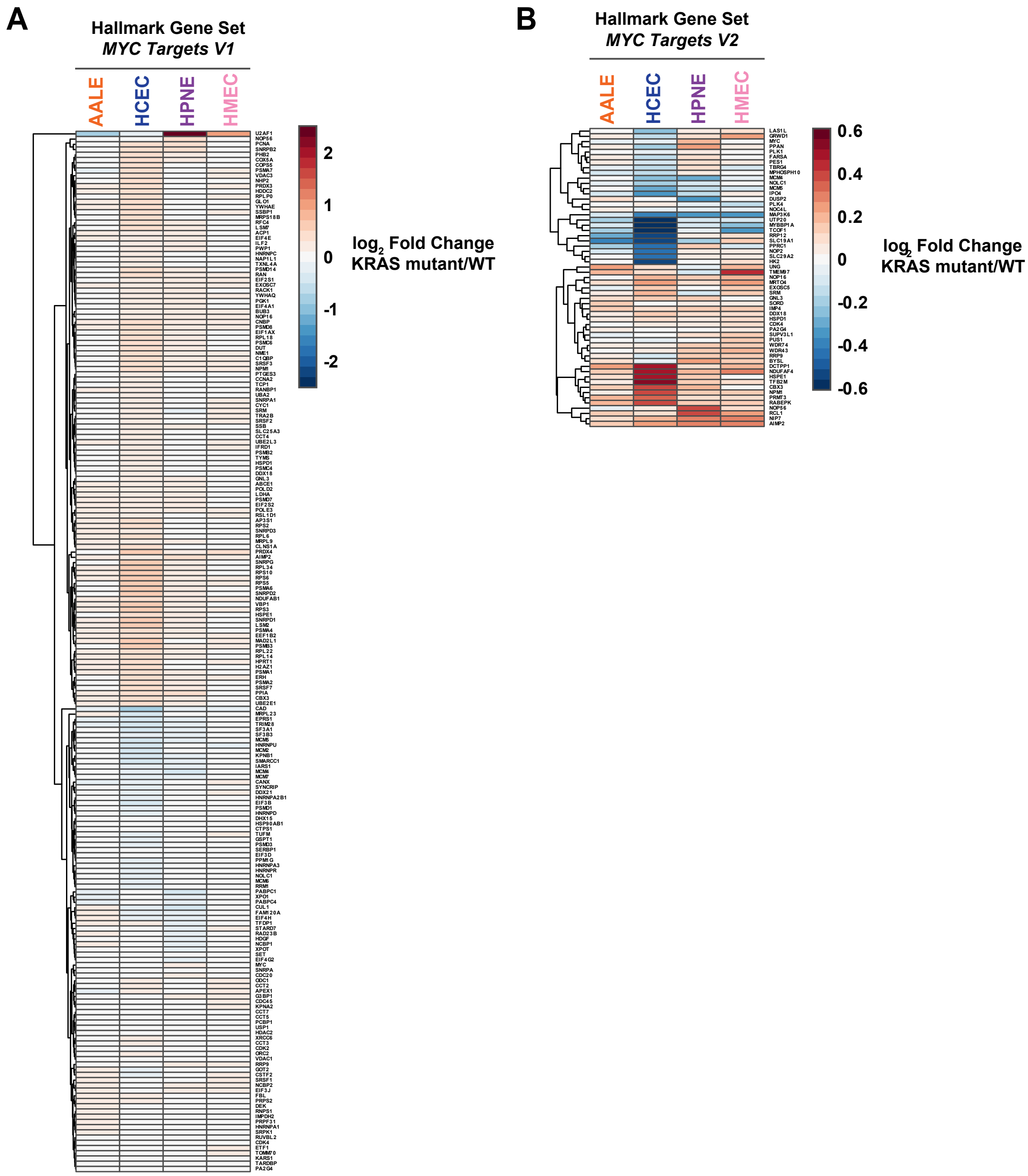

Figure S2. Comparison of MYC gene signature upregulation after mutant KRAS expression across isogenic cell lines. A. Comparison of MYC Hallmark V1 gene set expression changes after mutant KRAS expression across tissue lineages. **B.** Comparison of MYC Hallmark V2 gene set expression changes after mutant KRAS expression across tissue lineages.

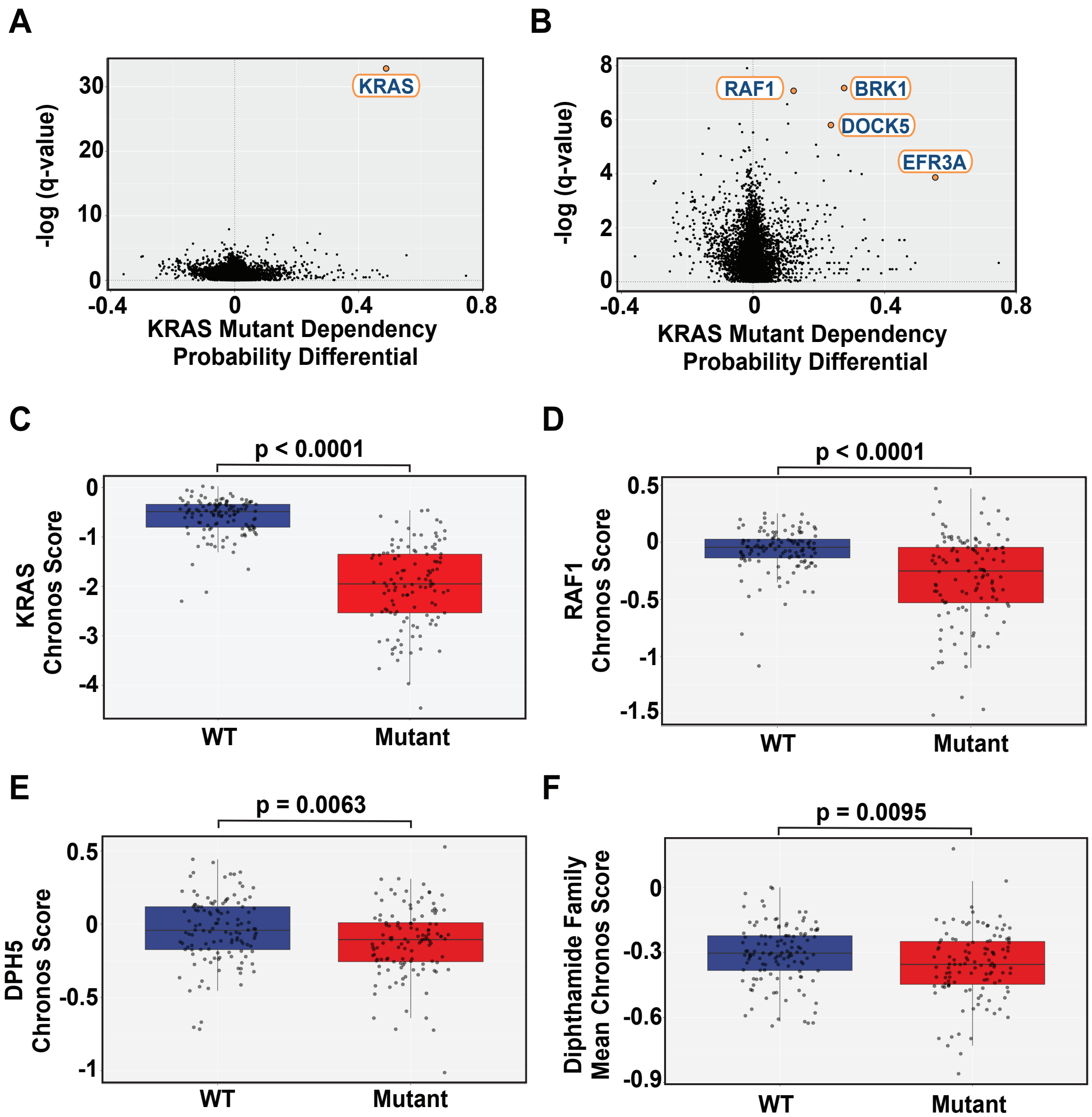

Figure S3. Analysis of KRAS mutant gene dependencies from the Cancer DepMap. A. Gene dependency differences in KRAS WT and mutant human colon, lung, and pancreatic tumor cells. Post-Chronos gene dependency probability estimates were compared between KRAS WT and KRAS mutant cell lines from whole-genome CRISPR screens. A differential of the probability estimate was determined by subtracting the KRAS WT probability from the KRAS mutant probability, where values closer to +1 indicate more essential in KRAS mutant cells and -1 indicate more essential in KRAS WT cells. **B.** Gene dependency plot after removing the KRAS gene itself to provide a zoomed-in view of KRAS mutant dependencies. **C.** Loss of KRAS is more toxic to KRAS mutant cell lines. **D.** Loss of c-Raf is more toxic in KRAS mutant cells. **E.** KRAS mutant cells are more sensitive to DPH5 knockout. **F.** KRAS mutant cells are more sensitive to inhibition of the diphthamide synthesis pathway.

Dataset S1. RNA-sequencing data and analyses from the isogenic KRAS cell lines. Raw and normalized read counts for each gene, DESeq2 analyses comparing *KRAS* WT to *KRAS* mutant cells, upregulated and downregulated genes (FDR < 0.05), and GSEA for each cell line.

Dataset S2. CRISPR screening data and analyses from the isogenic KRAS cell lines. Raw sgRNA read counts, MAGeCK and edgeR analyses for KRAS WT and KRAS mutant CRISPR screens, KRAS SL gene overlaps between tissue lineages, and GSEA of KRAS SL genes for each cell line.

Dataset S3. Cancer Dependency Map (DepMap) analysis to identify putative KRAS SL genes across lung, colon, and pancreas cells. Gene dependency scores for KRAS WT and KRAS mutant cells and adjusted p-values based on whole-genome CRISPR screens.
